# Aneurysmal subarachnoid haemorrhage: effect of CRHR1 genotype on fatigue and depression

**DOI:** 10.1186/s12883-020-01727-y

**Published:** 2020-04-18

**Authors:** Artur Vetkas, Ele Prans, Sulev Kõks, Tõnu Rätsep, Toomas Asser

**Affiliations:** 1 Tartu University Hospital, Tartu, Estonia; 2 Tartu University, Tartu, Estonia; 3 Centre for Molecular Medicine and Innovative Therapeutics, Murdoch University, Perth, WA Australia; 4 The Perron Institute for Neurological and Translational Science, Perth, WA Australia

**Keywords:** Aneurysm, Subarachnoid haemorrhage, Fatigue, Depression, Mental health, Cortitrophin-releasing hormone receptor-type 1, CRHR1, Gene, SNP

## Abstract

**Background:**

Emotional health disturbances are common after aneurysmal subarachnoid hemorrhage (aSAH) and their causes are largely unexplored. Corticotropin-releasing hormone receptor 1 (CRHR1) is a key factor in stress reactivity and development of mental health disturbances after adverse life-events.

**Methods:**

We explore the effect of CRHR1 genotype on mental health after aSAH in a retrospective cohort study. One hundred twenty-five patients have been assessed using EST-Q mental health questionnaire. Genotyping of CRHR1 single nucleotide polymorphisms (SNP-s) was performed (Rs7209436, Rs110402, Rs242924).

**Results:**

Fatigue was present in almost half of aSAH patients, depression and anxiety in one-third. There was a high prevalence of insomnia and panic complaints. Rs110402 minor allele decreased the risk of depression (OR = 0.25, *p* = 0.027 for homozygotes). Depression was present in 14% vs 41% in minor and major allele homozygotes, respectively. Rs110402, Rs242924 and Rs7209436 minor alleles and TAT-haplotype, formed by them, were protective against fatigue. After Bonferroni correction only the association of Rs110402 with fatigue remained statistically significant (OR = 0.21, *p* = 0.006 for minor allele homozygotes). Results remained statistically significant when adjusted for gender, admission state, age and time from aSAH. In multiple regression analysis occurrence of fatigue was dependent on anxiety, modified Rankin score and Rs110402 genotype (R^2^ = 0.34, *p* <  0.001).

**Conclusions:**

CRHR1 minor genotype was associated with a lower risk of fatigue and depression after aSAH. Genetic predisposition to mental health disturbances associated with negative life-events could be a risk factor for fatigue and depression after aSAH and selected patients might benefit from advanced counselling in the recovery phase.

## Background

Incidence of aneurysmal subarachnoid haemorrhage (aSAH) is around 7.9 per 100,000 patient years [1]. Up to 65% survive the subacute phase and two-thirds of those patients recover to functional independence [2, 3]. Only 15% develop a focal neurological deficit or need assistance in ambulation [4]. At the same time, many aSAH patients experience emotional and cognitive problems - anxiety and depression occurs in up to half of aSAH cases, post-traumatic stress disorder (PTSD) is reported in up to a third and occasional or constant fear of recurrent bleeding in two-thirds of patients [5, 6]. Decrease in health-related quality of life is a well-documented sequela of aSAH and changes in mental component of quality of life were recently associated with CRHR1 genotype [3, 7].

The amount of emotional disturbances after aSAH seems to be out of proportion to cognitive and neurological impairments and causes of these changes are largely unexplored. Genetic predisposition might have a role in the formation of psychological disturbances after aSAH. The hypothalamic–pituitary–adrenal (HPA) axis response to stress is moderated by genetic and environmental factors and is implicated in pathogenesis of emotional diseases [8]. Genes that regulate the function of the stress response system are probable moderators of the effect that adverse life events have on the development of emotional illness [9].

Corticotropin-releasing hormone (CRH) is a key stress mediator in the central nervous system [10]. Corticotropin-releasing hormone receptor 1 (CRHR1) is central in activating mesolimbic and HPA responses to different types of stress. CRHR1 genotype has been associated with mental health disorders and response to antidepressant treatment [11]. It has been reported to moderate the effects of stressful life events on development of major depression and moderate stress related cortisol reactivity [12, 13]. Cortisol response to stress has been associated with depression and anxiety symptoms [14]. An elevated cortisol response is generally associated with major depression (MD) [15], and a flattened cortisol response is associated with panic disorder and post-traumatic stress disorder (PTSD) [16].

Few genetic studies have been conducted on the topic of neuropsychological disturbances after aSAH [16]. The objective of current research was to characterize specific neuropsychiatric disturbances after aSAH and explore the influence of CRHR1 genotype on their occurrence.

## Methods

**Fig. 1:**
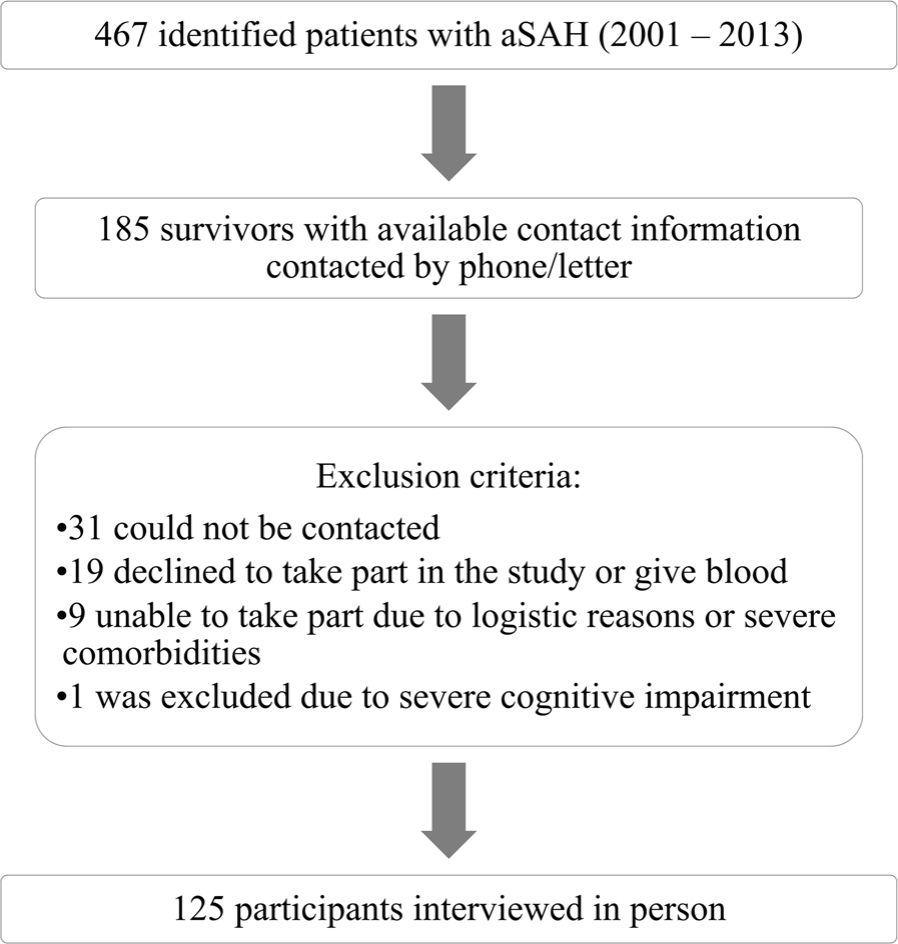
Selection of patients

We performed a retrospective cohort study of long-term outcome of 125 surgically treated aSAH survivors. The studied group was selected from medical records of all patients diagnosed with aSAH (*n* = 467) from January 2001 to November 2013 in a university clinic (spontaneous ICH and other SAH causes were excluded). Selection criteria are presented in Fig. 1.

The average age at ictus was 54 years (SD = 13; range 24–82 years). The average time between initial hospitalisation and the study was 4 years (SD = 2.8; range 1–13 years). 41% (*n* = 51) of the patients were studied more than 3 years from ictus. 70% (*n* = 88) of the patients were female. 78% (*n* = 88) of the patients had more than 10 years of education. One patient was diagnosed with depression prior to aSAH based on the national database information available from 2009. The patients were interviewed in person with a structured questionnaire and blood samples were gathered. All participants gave their written informed consent and ethical approval was received from the institutional ethics committee.

### General management of patients

All aSAH patients were managed according to general guidelines and our protocol is previously published [17]. All patients were admitted acutely and SAH was diagnosed by computer tomography (CT) or lumbar puncture. The aneurysm was assessed by CT-angiography or digital subtraction angiography. All patients were initially managed in a neurointensive care unit. Almost all patients were acutely operated upon, preferably via a pterional approach, and the aneurysms were clipped using standard microsurgical techniques. Endovascular procedures were preferentially performed in a separate institution during this time. Due to this our study includes a series of clipped patients. Clinical data of the patients and complications are presented in Table 1.

**Table 1:** Characteristics of aSAH, complications and clinical outcome

Characteristic	N (%)
Male	37 (30%)
Female	88 (70%)
Hunt Hess score	
1	17 (14%)
2	66 (53%)
3	23 (18%)
4	14 (11%)
5	5 (4%)
Aneurysm location	
ICA	40 (32%)
AcomA	44 (35%)
MCA	22 (18%)
ACA	8 (6%)
BA	9 (7%)
VA	2 (2%)
Intracerebral haemorrhage	22 (18%)
Symptomatic vasospasm	34 (27%)
Ischemic lesions on CT	25 (20%)
Hydrocephalus	
acute	43 (34%)
chronic	14^a^ (11%)
Modified Rankin Score	
0	4 (3%)
1	7 (6%)
2	57 (46%)
3	49 (39%)
4	8 (6%)

Abbreviations: *ICA* internal carotid artery, *AcomA* anterior communicating artery, *MCA* middle cerebral artery, *ACA* anterior cerebral artery, *BA* basilar artery, *VA* vertebral artery, *CT* computer tomography. ^a^These 14 patients required ventriculoperitoneal shunting after aSAH

### Procedure

Clinical data and Hunt and Hess grade (HH) [18], which is a clinical grading system designed to predict prognosis and outcome in aSAH, were recorded at ictus. Remaining data was collected during the follow-up evaluation, when the patients were interviewed in person with a structured questionnaire (Additional file 1). Patient clinical recovery was evaluated according to the modified Rankin Scale (mRS) during the clinical interview [19]. Patients were also questioned about treatment for emotional disorders after aSAH, comorbidities, education and social living situation (living with family/someone else or alone). Emotional State Questionnaire (EST-Q) was used to measure emotional health.

EST-Q is a well-validated self-rating scale that contains scales of Depression, Anxiety, Agoraphobia-Panic, Fatigue and Insomnia [20]. The items of EST-Q were derived from diagnostic criteria of DSMIV and ICD-10. Each item is rated by occurrence on a five-point scale ranging from 0 to 4 (respectively ‘not’ and ‘all the time’). According to the results of factor analysis, EST-Q scales have demonstrated internal consistency of 0.69–0.88 Cronbach alpha. The participants were asked to report how much the various problems troubled them during the past 4 weeks. Scoring more than the cut-off point in a specific scale shows that the scale score is in the same magnitude as that of most patients with the given medical condition. The cut-off points for clinically important symptomatology were ≥ 12 points for depression and anxiety, ≥ 8 points for fatigue, ≥ 7 points for agoraphobia–panic and ≥ 6 points for insomnia [21]. EST-Q includes a question about frequency of ‘Recurrent thoughts of death or suicide’. We have described the methodology of EST-Q in more detail previously [17]. The data was compared with an age and gender matched general population of 3923 subjects (obtained from the 6434 respondents of a National Health Interview Survey).

### Genotyping

The genomic DNA was extracted from venous blood samples in 4 ml EDTA containing vaccuettes by using the standard salting-out method. The EDTA tubes were stored at -20C until DNA extraction. Isolated DNA was dissolved in Tris-EDTA (TE) buffer. The purity and concentrations of the DNA were measured by a spectrophotometer (NanoDrop, ND-1000). The gDNA samples were aliquotted and stored at -80C until usage. Genotyping of marker single nucleotide polymorphisms (SNP) Rs7209436, Rs110402, Rs242924 and Rs242939 was carried out by using TaqMan SNP Genotyping Assay (Applied Biosystems, Foster City, CA, USA), which is a multiplex endpoint assay that detects variants of a single nucleic acid sequence. PCR reactions were run on the ViiA7 instrument (Applied Biosystems, Foster City, CA, USA) by using the following cycling parameters: after the first step at 95C for 10 min, 40 cycles of denaturation at 92C for 15 s and extension at 60C for 1 min. Genomic DNA (20 ng/ul) was amplified in a total volume of 10ul containing 1x Amplification Master Mix (Applied Biosystems, Foster City, CA, USA) and 1x probe. Genotypes were analysed by using the allelic discrimination function of the system (Table 2). There was no statistically significant difference between minor and major CRHR1 genotype with sociodemographic characteristics.

**Table 2:** CRHR1 allele distribution (*n* = 125)

SNP	Genotype (n)			Minor allele (n)	Major allele (n)
Rs7209436	C/C (34)	C/T (69)	T/T (22)	T (91)	C (103)
Rs110402	G/G (29)	A/G (68)	A/A (28)	A (96)	G (97)
Rs242924	G/G (31)	G/T (67)	T/T (27)	T (94)	G (98)

Abbreviations: *CRHR1* corticotropin-releasing hormone receptor 1, *SNP*single nucleotide polymorphism

### Statistics

Student’s t-test was used to compare patients and age and gender matched general population EST-Q scores and determine the associations with clinical or sociodemographic factors. All continuous variables were controlled for normality using Shapiro-Wilk’s W test. Logistic regression analysis was performed to study the association of CRHR1 genotype and EST-Q scores and calculate odds ratios (OR). EST-Q scores were used as indicator variables characterizing whether the individual possesses the emotional disturbances or not according to the cut-off values. In the SNP analysis we chose between additive/dominant/recessive model based on the AIC (Akaike information criterion) of the unadjusted model (recessive model – minor allele homozygote, dominant model – major allele homozygote, additive model – OR for heterozygotes, which is multiplied in case of minor allele addition (minor homozygote – OR x OR)). When recessive genotype was rare (3 or less patients) then dominant model was preferred. Results were considered significant if *p* <  0.05. The *p*-values that survived the Bonferroni correction are marked in bold. Pearson’s correlation was used to asses internal correlation in EST-Q scales and logistic regression analysis was used to describe the influence of CRHR1 genotype (frequency of minor alleles) and patient factors on fatigue. Statistical analysis was performed with Stata 14.2 (StataCorp LLC) and SPSS 24 (IBM). Statistical analysis was performed with R (The R Foundation) and Stata 14.2 (StataCorp LLC).

## Results

The average age at follow-up was 58 years (SD = 12, range 26–82). 78% (*n* = 97) of the patients were living with family or someone else. 67% (*n* = 84) had hypertension, 14% (*n* = 18) joint pain or rheumatoid arthritis, 7% (*n* = 9) had diabetes and 2% (*n* = 3) reported myocardial infarction. 24% (*n* = 30) of the patients received metoprolol, 17% (*n* = 21) amlodipine, 11% (*n* = 14) ramipril, 8% (*n* = 10) telmisartan, 6% (*n* = 8) enalapril, and 6% (*n* = 8) received perindopril/indapamide. Other medications were less common. 41% (*n* = 51) of the patients required help in everyday life. 24% (*n* = 30) of the patients saw a psychologist or psychiatrist and 38% (*n* = 47) used antidepressants or similar medication at some point after aSAH.

Of the patients, 55% (*n* = 68) had a mRS score of 0–2 and 39% (*n* = 48) had a score of 3. Worse mRS score was associated with female gender (2.5 (SD = 0.8) vs 2.1 (SD = 0.8), *p* = 0.019) and requiring more daily help (3.1 (SD = 0.5) vs 2.0 (SD = 0.7), *p* <  0.001).

### Emotional outcome scores

Patients scored significantly worse on all EST-Q scales compared to the general population (Table 3). Female gender was associated with worse EST-Q scores: depression (mean 9 (SD = 6.8) vs 5.6 (SD = 4.7), *p* = 0.006), anxiety (mean 9.2 (SD = 5.8) vs 6.7 (SD = 4.9), *p* = 0.021), agoraphobia-panic (mean 3.8 (SD = 4.3) vs 1.2 (SD = 2), *p* <  0.001), fatigue (mean 7.2 (SD = 4.2) vs 5.4 (SD = 4), *p* = 0.03), and insomnia (mean 5.1 (SD = 3.3) vs 3.7 (SD = 3.6), *p* = 0.04). A worse fatigue score was associated with having hypertension (mean 7.5 (SD ± 4.2) vs 4.9 (SD ± 3.6), *p* <  0.001) and having joint pains (mean 8.6 (SD ± 3.4) vs 6.4 (SD ± 4.3), *p* = 0.021). Taking amlodipine was associated with a worse depression score (10.7 (SD ± 6.9) vs mean 7.5 (SD ± 6.3), *p* = 0.035).

**Table 3:** EST-Q scores among aSAH patients (*n* = 125) and age and gender matched general population (*n* = 3923)

EST-Q scales	aSAH mean, *n* = 125	aSAH SD	Population mean, *n* = 3923	Population SD	*p*-value
Depression	8	6.5	4.3	5.4	< 0.001
Anxiety	8.5	5.6	4.5	4.4	< 0.001
Agoraphobia-Panic	3	3.9	0.9	2.6	< 0.001
Fatigue	6.7	4.2	3.1	2.7	< 0.001
Insomnia	4.7	3.4	3.2	3	< 0.001

Abbreviations: *SD* standard deviation, *p p*-value

**Fig. 2:**
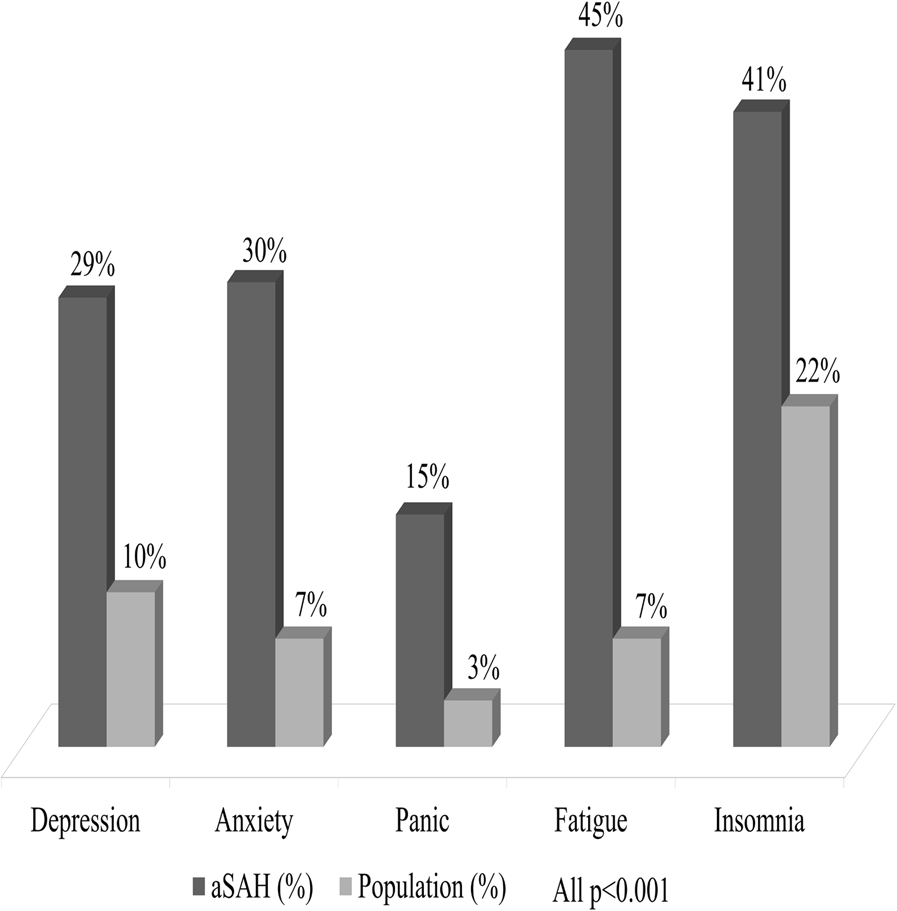
Prevalence (%) of emotional disorders according to cut-off values among aSAH patients (*n* = 125) and age and gender matched general population (*n* = 3923) based on the EST-Q cut-off scores (all *p* < 0.001)

aSAH patients had a high prevalence of those scoring above the cut-off values for emotional disturbances compared to the general population, with fatigue and insomnia being the most common problems presenting in around half of the patients (Fig. 2). About one third of the patients had higher than cut-off scores demonstrating depression (29%, *n* = 36) and anxiety (30%, *n* = 38). 15% of patients (*n* = 19) had higher than cut-off values on agoraphobia-panic scale. 14% (*n* = 18) of aSAH patients had frequent thoughts of death or suicide (ranging from sometimes to constant) compared to 3% (*n* = 126) in the general population (*p* <  0.001).

### Association of genotype with EST-Q outcomes

In regression analysis we explored the association of CRHR1 genotype with emotional disorder symptoms (Table 4). The results remained significant after adjustment for gender, neurological state at admission (HH), patient age and time from aSAH to evaluation. Effect of Rs110402 on fatigue remained statistically significant after Bonferroni correction for multiple comparisons.

**Table 4:** Influence of genotype on EST-Q outcomes (only statistically significant results are reported)

SNP	Allele	Model	OR	95% CI	p	OR*	95% CI*	p*
**Depression**								
Rs110402	Minor	Additive	0.50	0.28–0.92	0.027	0.50	0.26–0.94	0.032
**Fatigue**								
Rs110402	Minor	Additive	0.46	0.26–0.8	**0.006**	0.48	0.27–0.85	0.012
Rs7209436	Minor	Recessive	0.22	0.07–0.69	0.009	0.23	0.07–0.75	0.015
Rs242924	Minor	Recessive	0.35	0.14–0.9	0.030	0.37	0.14–0.98	0.044
TAT-haplotype	Minor	Additive	0.26	0.08–0.81	0.021	0.27	0.08–0.88	0.030
**Insomnia**								
Rs242939	Minor	Additive	0.43	0.18–1.02	0.057	0.38	0.15–0.96	0.042

Abbreviations: *OR* odds ratio, *CI* confidence interval. *P*-values that are adjusted for gender, Hunt Hess score, patient age and time of evaluation from aSAH are marked with *. *P*-values that survived the Bonferroni correction are marked with bold

**Fig. 3:**
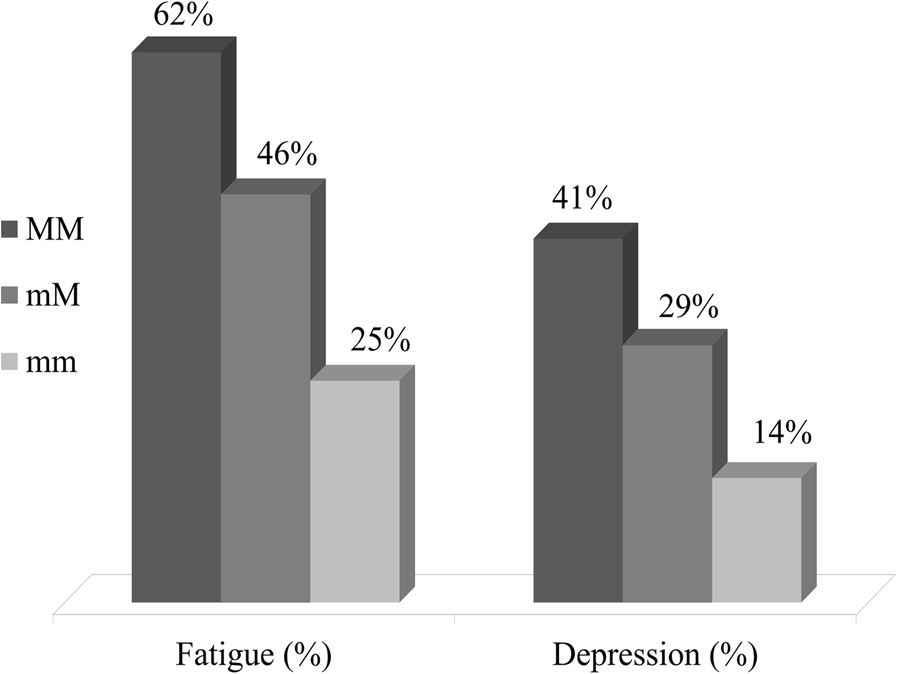
Prevalence of fatigue and depression based on cut-off values in aSAH patients (*n* = 125) according to Rs110402 to genotype. MM – homozygote for major allele, mM – heterozygote, mm – homozygote for minor allele

Rs110402 minor genotype carriers had a reduced risk of depression (OR = 0.5, 95% CI, 0.28–0.92, *p* = 0.027) in the additive model. Among patients with the minor Rs110402 alleles prevalence of depression was close to the general population (14%) and much lower than in patients with the major genotype (41%) (Fig. 3).

CRHR1 minor alleles (Rs7209436, Rs110402, Rs242924) had a protective effect against developing fatigue (OR = 0.22–0.46, *p* <  0.05) in additive and recessive models. Prevalence of fatigue was 62% vs 25% in major and minor allele homozygotes of Rs110402, respectively (Fig. 3). TAT haplotype, formed by the three minor alleles, was associated with a significant protective effect against fatigue (OR = 0.26, 95% CI, 0.08–0.81, *p* = 0.021) in the additive model.

Rs242939 minor allele had a protective effect against insomnia when adjusted for gender, Hunt Hess score, patient age and time of evaluation from aSAH (OR* = 0.43, 95% CI, 0.18–1.02, p* = 0.057) in the additive model (Table 4).

### Internal correlation and factors influencing fatigue

Almost half of aSAH patients had higher than cut-off EST-Q scores indicating fatigue (45%; *n* = 56) and insomnia (41%; *n* = 51). Mental health disorders, as diagnosed by EST-Q cut-off values, were significantly correlated with each other (Table 5). There was a moderate correlation between mRS and EST-Q scales, with higher values for fatigue and depression. Rs110402 was significantly correlated to fatigue and depression and Rs7209436 to fatigue.

**Table 5:** Correlations between EST-Q scales and CRHR1 genotype in aSAH patients (*n* = 125). *p < 0.001; **p < 0.01; ***p < 0.05

Variables	Fatigue	Depression	Anxiety	Panic-Agorophobia	Insomnia	mRS	Rs110402	Rs7209436
**Depression**	0.42*							
**Anxiety**	0.49*	0.54*						
**Panic-Agorophobia**	0.34*	0.47*	0.59*					
**Insomnia**	0.33*	0.33*	0.34*	0.28**				
**mRS**	0.4**	0.34**	0.29**	0.28**	0.23**			
**Rs110402**	−0.25***	−0.20***	−0.12	−0.06	−0.06	−0.14		
**Rs7209436**	−0.23***	− 0.15	−0.12	− 0.06	−0.1	− 0.14	0.89***	
**Rs242924**	−0.15	−0.15	− 0.07	−0.01	− 0.06	−0.16	0. 86***	0.9***

We performed a multiple regression analysis for fatigue due to its reported association with anxiety and depression. The best model for fatigue included anxiety, mRS and Rs110402 genotype (number of minor alleles) with a R^2^ = 0.34, *p* <  0.001 (Table 6). In a separate analysis, with insomnia entered instead of mRS, the model still had good explanatory value, but R^2^ decreased to 0.31, *p* <  0.001. Sociodemographic factors and comorbidities were not associated with fatigue in multiple regression analysis.

**Table 6:** Multiple regression for fatigue occurrence based on cut-off values

Variables	B	SE	β	***p***-value
Anxiety	0.42	0.08	0.39	< 0.001
mRS	0.016	0.05	0.27	0.001
Rs110402	−0.12	0.06	−0.17	0.027

## Discussion

We describe an association of CRHR1 genotype with mental health after aSAH. Multiple logistic regression analysis showed that corticotropin-releasing hormone receptor 1 genotype (Rs7209436, Rs110402, Rs242924) was associated with mental health disorders measured with EST-Q. Rs110402 minor allele decreased the risk of depression (OR = 0.25, *p* = 0.027 for minor allele homozygotes). Rs110402, Rs242924 and Rs7209436 minor alleles and TAT-haplotype formed by them were protective against developing fatigue (OR = 0.22–0.46, *p* <  0.05). Only association of Rs110402 with fatigue remained statistically significant after Bonferroni correction (OR = 0.21, *p* = 0.006 for minor allele homozygotes). Rs242939 minor genotype was associated with a lower prevalence of insomnia in the adjusted model.

Our study shows that aSAH patients frequently experience long-term mental health disturbances. Emotional disturbances have been reported after stroke. A recent study described similarities among patient perceived outcomes in quality of life components after different types of stroke (ischemic stroke, intracerebral hematoma, and SAH) [22]. These patients, similar to aSAH, experience a severe life-changing event, which requires habituation and acquiring a new social role. Post-stroke depression occurs in up to 33% and anxiety in up to 25% of cases, with reported associations between them [23, 24]. In our study almost 1/3 of aSAH patients scored above the cut-off values of EST-Q indicating depression and anxiety. 20% (*n* = 25) showed a coexistence of the two conditions. Depression and anxiety were moderately correlated with each other (Pearson’s *r* = 0.54, *p* < 0.001) and presented more often among women.

Almost half of the patients in our study (45%) scored above the cut-off value for fatigue. Fatigue is considered to be the most common complaint after stroke and it occurs in up to 50% of cases [25]. Fatigue could be defined as “a state characterized by weariness unrelated to previous exertion levels and usually not ameliorated by rest” [26]. Fatigue is considered to be a separate entity from other mental health disorders and its occurrence after neurological diseases is more frequent than would be expected based on age and disability [27]. Among younger patients with stroke, fatigue predicts a worse health-related quality of life than neurologic dysfunction [28]. Fatigue has a detrimental effect on quality of life by restricting everyday activities and it can persist due to a state of mental exhaustion from having to deal with processes of rehabilitation and adaptation. Poststroke fatigue has been associated with anxiety and depression, but it can occur separately from them [29]. Fatigue can persist after successful treatment of major depression and it requires distinct interventions [30]. In our study fatigue showed a moderate correlation to anxiety (Pearson’s *r* = 0.49, *p* < 0.001) and depression (*r* = 0.42, *p* < 0.001). In a multiple regression model, 34% of variance in fatigue was best explained by anxiety, mRS score and Rs110402 genotype, *p* < 0.001). This implies that fatigue is related both to physical and mental complaints of the patients and requires a multidisciplinary management.

We describe a significant number of patients reporting thoughts of death and suicide (14%, *n* = 18), which reflects on the extent of their emotional problem. Suicidality was not associated with CRHR1 genotype in our study. Patients also had a high prevalence of agoraphobia-panic complaints and twice as much sleep problems as the general population. 70% of the patients in our study were female and female gender was associated with worse EST-Q scores.

The neural events of mental health disturbances after aSAH, measured in this study with EST-Q, remain largely unknown. Hormonal changes have been proposed as a cause, but studies reported varying results [31]. Coping strategies and premorbid psychiatric history could affect the long-term outcome of patients [32], but are hard to study retrospectively. It is possible that patients have a psychological reaction to a sudden traumatic event and live with a recurrent fear of rebleeding that affects their emotional well-being [6]. Patients might be stigmatised by the disease or there is a distinct neurobiological predisposition for psychological maladaptation after aSAH. Understanding that a person with an intrinsic vulnerability is at more risk of developing a mental illness or an inadequate psychological reaction to a stressful life event is well acknowledged in the psychiatric community [33].

It is unknown whether the studied SNP-s are functional or they are in linkage disequilibrium with other regions, but it has been previously reported that CRHR1 genotype is related to formation of major depression after stressful life events and could moderate cortisol reactivity to stress [12, 13]. In a multilocus genetic profile score study, CRHR1 together with other HPA genes has been shown to interact with chronic stress moderating diurnal cortisol slope and predicting fatigue [34]. CRHR1 genotype has been associated with PTSD and depression symptoms in survivors of post-surgical intensive care unit treatment [35]. Cortisol dysbalance occurs in critical illness patients, with a possible insufficiency after aSAH [36]. Corticosteroid administration was associated with a decreased frequency of post intensive care PTSD symptoms after major surgery [37].

Emotional health disturbances after aSAH are reported rather unhomogenously. Some studies describe the patients as being depressed, having anxiety, chronically fatigued or experiencing PTSD symptoms. Psychiatric disturbances have a certain overlapping and a more uniform diagnostic strategy is required. There are few genetic studies related to outcomes after aSAH and their results have been inconclusive. One of the shortcomings is that outcomes were mostly measured with general scoring systems (Glasgow Outcome Scale, modified Rankin Scale), which are clinically relevant, but do not reflect on the entire state of patients recovery [5]. Health related quality of life was assessed separately in the same group of aneurysmal SAH patients using SF-36 questionnaire and it was reduced in all domains including mental health, where it was positively associated with minor CRHR1 genotype [7]. The strength of the current study is that patients were characterised using a sensitive assessment tool that showed an array of psychological symptoms with high prevalence in post-aSAH patients, including suicidal thoughts. To the best of our knowledge, this is the first study reporting a genetic association with specific mental health disturbances after aSAH. The different emotional problems that aSAH patients experience could be interconnected, but they most likely require distinct neurocognitive interventions and counselling. Mental health disturbances could interfere with patients returning to work and increase the disease burden [38, 39]. We describe a neurobiological predisposition for development of depression and fatigue after aSAH which could help in selecting patients at risk and potentially guiding their therapy.

### Limitation of the study

Our study was a retrospective design and included 125 patients, due to this our results in SNP analysis might be underpowered to draw certain conclusions. Despite mental health diagnosis being extractable from the national database, we don’t know the full extent of previous emotional problems in our patient group. Cultural factors could also influence outcomes. We lack information regarding the cognitive profile of the patients, but none of them had severe disabilities when interviewed. Our findings need to be validated in a larger prospective cohort and further studies are required to determine the functional consequences of CRHR1 genotype variability.

## Conclusions

Results of our study suggest that prevalence of long-term emotional health disorders after aSAH is high with depression and anxiety presenting in one-third of patients. Fatigue is the most common problem and it is influenced both by mental and physical state of the patients. CRHR1 minor genotype was associated with less fatigue and depression symptoms after aSAH. The association with CRHR1 could be due to its role in the HPA-system response to stress and predisposition of patients to emotional problems after adverse life-events. New and improved biomarkers are needed to predict, diagnose and treat the long-term consequences of aSAH. Such biomarkers could help identify patients who would benefit from early neuropsychological rehabilitation and decrease the disease burden.

## Supplementary information

**Additional file 1.** The file contains a description of the EST-Q questionnaire used to asses mental health disturbances and general questions used to asses the recovery process of patients.

## Data Availability

The datasets generated and/or analysed during the current study are not publicly available due to restrictions.

## Abbreviations

aSAH: Aneurysmal subarachnoid hemorrhage
CRHR1: Corticotropin-releasing hormone receptor
SNP: Single nucleotide polymorphism
PTSD: Post-traumatic stress disorder
HPA axis: Hypothalamic–pituitary–adrenal axis
MD: Major depression
SF-36: Short Form 36
CT: Computer tomography
mRS: Modified Rankin Scale
EST-Q: Emotional State Questionnaire
HH: Hunt and Hess grade
DSMIV: Diagnostic and Statistical Manual of Mental Disorders 4th Edition
ICD-10: International Classification of Diseases 10th Edition
DNA: Deoxyribonucleic acid
EDTA: Ethylenediaminetetraacetic acid
TE: Tris-EDTA
AIC: Akaike information criterion
ICA: Internal carotid artery
AcomA: Anterior communicating artery
MCA: Middle cerebral artery
ACA: Anterior cerebral artery
BA: Basilar artery
VA: Vertebral artery
SD: Standard deviation
p: *p*-value
OR: Odds ratio
CI: Confidence interval
