# Supplementary material for: Aneurysmal subarachnoid haemorrhage: effect of CRHR1 genotype on fatigue and depression
Source: BMC Neurol. 2020 Apr 18;20:142. doi: 10.1186/s12883-020-01727-y (PMC7165373; doi:10.1186/s12883-020-01727-y)
Supplement: Supplementary file 1 — Additional file 1. The file contains a description of the EST-Q questionnaire used to asses mental health disturbances and general questions used to asses the recovery process of patients. [file 12883_2020_1727_MOESM1_ESM.docx]

**Additional File 1**

1. Emotional state questionnaire used in the study has been developed elsewhere and previously published.

**EST-Q:**

Aluoja A, Shlik J, Vasar V, Luuk K, Leinsalu M. Development and psychometric properties of the Emotional State Questionnaire, a self-report questionnaire for depression and anxiety. Nord J Psychiatry. 1999;53:443–9. doi:10.1080/080394899427692.

EST-Q is self-rating scale that contains scales of Depression, Anxiety, Agoraphobia-Panic, Fatigue and Insomnia. The items of EST-Q were derived from diagnostic criteria of DSMIV and ICD-10. Each item is rated by occurrence on a five-point scale ranging from 0 to 4 (respectively ‘not’ and ‘all the time’). The participants were asked to report how much the various problems troubled them during the past 4 weeks. Scoring more than the cut-off point in a specific scale shows that the scale score is in the same magnitude as that of most patients with the given medical condition. The cut-off points for clinically important symptomatology were ≥ 12 points for depression and anxiety, ≥ 8 points for fatigue, ≥ 7 points for agoraphobia–panic and ≥ 6 points for insomnia.

Questions according to EST-Q scales:

**Depression**

1. Feelings of sadness

3. Feeling no interest or pleasure in things

5. Feelings of worthlessness

6. Self-accusations

7. Recurrent thoughts of death or suicide

16. Feeling lonely

17. Hopelessness about the future

18. Impossible to enjoy things

**Agoraphobia–panic**

25. Sudden attacks of panic with palpitations, shortness of breath, faintness, or other frightening bodily sensations

29. Fear of being outside home alone

30. Feeling afraid in streets or open places

31. Fear of fainting in public

32. Feeling afraid of travelling by bus, train, or car

**Anxiety**

2. Feeling easily irritated or annoyed

20. Feeling anxious or fearful

22. Tension or inability to relax

23. Excessive worry about several different things

24. Feeling so restless that it is hard to sit still

26. Easily startled

**Fatigue**

4. Fatigue or loss of energy

8. Diminished ability to think or concentrate

9. Feeling slowed down

19. Rest does not restore strength

21. Being easily fatigued

**Insomnia**

10. Difficulty falling asleep

11. Restless or disturbed sleep

12. Waking up too early

1. General questions about patient’s health and social living situations that have been asked:

- Other disease or health problems that you have:

1) □ Hypertension

2) □ Ischemic heart disease

3) □ Myocardial infarction

4) □ Previous stroke

5) □ Diabetes

6) ……………………………..

- Do you have any health complaints or disease that reduce your quality of life?

Please name them:

……………………………………………………………………………………………..

- Please state you living situation:

□ Living alone

□ Living with family

□ Living with somebody else

□ Other ………………………..

- Did you return to work after recovering from aneurysmal subarachnoid haemorrhage?

□ Yes

□ No

- Do you work now?

□ Yes

□ No

- What is your level of education?

………………………………

- What specific treatment did you use after aneurysmal subarachnoid haemorrhage?

□ Antidepressants

□ Psychologist/psychiatrist

□ Physiotherapy

□ Painkillers/analgetics

□ Other ……………………………

- Do you feel that you completely recovered from aneurysmal subarachnoid haemorrhage?

□ Yes

□ No

Do you require help with everyday activities?

□ Yes

□ No
